# Molecular Detection of *Leishmania* spp. in Skin and Blood of Stray Dogs from Endemic Areas of Cutaneous Leishmaniasis in Saudi Arabia

**Published:** 2019

**Authors:** Abdullah D. ALANAZI, Robert PUSCHENDORF, Mohamed S. ALYOUSIF, Mohamed S. Al-KHALIFA, Samir A. ALHARBI, Zafer S. AL-SHEHRI, Ashraf E. SAID, Ibrahim O. ALANAZI, Hamdan I. AL-MOHAMMED, Yasser A. ALRAEY

**Affiliations:** 1. Department of Biological Science, Faculty of Science and Humanities, Shaqra University, Ad-Dawadimi, Saudi Arabia; 2. School of Biological Sciences, Plymouth University, Drake Circus, Plymouth, UK; 3. Department of Zoology, College of Science, King Saud University, Riyadh, Saudi Arabia; 4. National Center for Genomic Technology, King Abdulaziz City for Science and Technology, Riyadh, Saudi Arabia; 5. Department of Parasitology, Faculty of Medicine, King Faisal University, Al Ahsa, Saudi Arabia; 6. Department of Parasitology, Liverpool School of Tropical Medicine, Liverpool, UK

**Keywords:** *Leishmania*, Dogs, kDNA, Saudi Arabia

## Abstract

**Background:**

Dogs can act as reservoirs of canine leishmaniasis, caused by *Leishmania* species. The aims of this study were to determine the prevalence of canine leishmaniasis using a PCR technique among stray dogs living in three provinces of Saudi Arabia, Riyadh, Al-Ahsa Oasis and Al-Qaseem, where the disease is endemic; and to identify and document different *Leishmania* to species levels

**Methods:**

This cross-sectional investigation was conducted, from Mar 2016 to Apr 2018, in three parts of Saudi Arabia: Central province (Riyadh), Eastern province (Al-Ahsa Oasis) and Al-Qaseem province. Blood samples were collected from 526 dogs; 40 presented cutaneous nodules so were suspected clinically of cutaneous leishmaniasis. Biopsy tissue collections and parasite cultures were performed. A generic kDNA was performed using different primers for *Leishmania* differentiation.

**Results:**

All blood samples were negative for *Leishmania infantum* infection by molecular analysis, though forty dogs had thick cutaneous lesions in different parts of their body. Four dogs’ skin lesions were associated with dermatitis, splenomegaly and lymphadenomegaly. Parasite culture was used to diagnose cutaneous leishmaniasis, identifying 31/40 (77.5%) positive samples. Overall, of 526 samples, the prevalence of *L. major* and *L. tropica* was found to be 4% and 1.9%, respectively. Gender and age had a significant effect on *Leishmania* prevalence: (*P*=0.0212 and 0.0357), respectively.

**Conclusion:**

This was the first molecular study of dog leishmaniasis from Saudi Arabia of dogs confirmed to have cutaneous leishmaniasis. Further epidemiological and molecular investigations of domestic and wild canine infections with *L. major*, *L. tropica* and *L. infantum* in endemic and nonendemic areas of Saudi Arabia are required, for leishmaniasis control.

## Introduction

*L*eishmaniasis is a disease complex of various clinical manifestations caused by protozoan parasite infection (*Leishmania* spp.), transmitted through infected female sandfly bites (*Phlebotomus* spp. or *Lutzomyia* spp.) (1). This widespread disease was reported as one of the nine parasitic infections by WHO and is commonly seen in tropical and subtropical countries (2, 3). Epidemiological studies in the Middle East showed that anthroponotic cutaneous leishmaniasis caused by *L.tropica* and zoonotic cutaneous leishmaniasis caused by *L. major* occur in Saudi Arabia, Iraq, Iran, Afghanistan, Pakistan and Yemen (4–6), while visceral leishmaniasis caused by *Leishmania infantum* is reported in many countries such as Saudi Arabia, Iran, Afghanistan, Egypt, Iraq, and Yemen (5–7).

In Saudi Arabia, cutaneous leishmaniasis (CL) is endemic in different regions (8). The anthroponotic CL - caused by *L tropica,* transmitted by *Ph. sergenti -* is more endemic in the southwest and northwest, whereas *L. major* that causes zoonotic CL (ZCL) - transmitted by sand fly *Ph. papatasi,* possible reservoir host *Psammomys obesus* - is more seen in central and eastern regions like Al-Ahsa, Riyadh and Al-Qaseem (9–12). Visceral leishmaniasis (VL) is only reported in southwest Saudi Arabia (13–14).

Infected dogs are potential primary reservoirs (15). Cases of cutaneous and visceral infections caused by *L. tropica* were reported in Iran and Israel (16–18). Additionally, cases of cutaneous infection in *L. major* infected dogs have been reported in Saudi Arabia, Egypt, Iraq and Israel (19–22). In Saudi Arabia, although stray dogs have presented with clinical disease associated with *Leishmania* species infection, there is a lack of information on leishmaniasis, particularly in molecular levels. Most previous studies were epidemiological, clinical, histopathological and biochemical (23,24). Since dogs can be infected by sand flies in endemic areas, infected stray dogs may be a mobile risk for nonendemic surrounding areas (25).

Therefore, the objectives of this study were to detect the prevalence rates of CL and VL based on conventional (PCR) among stray dogs in three endemic Saudi provinces - Riyadh, Al-Ahsa Oasis and Al-Qaseem - and to identify and document different *Leishmania* at species level using a PCR technique.

## Materials and Methods

### Study areas

**Fig. 1:**
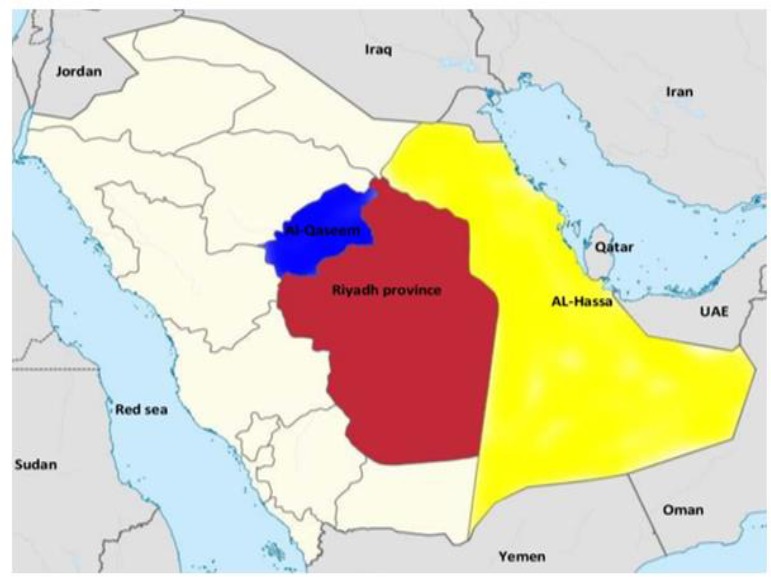
Map of the study area locations in Saudi Arabia

The investigation was conducted from Mar 2016 to Apr 2018 in three parts of Saudi Arabia; central province (Riyadh), Eastern province (Al-Ahsa Oasis) and Al-Qaseem province (Fig. 1). Stray dogs were trapped by live bait straps and selected randomly. A filed examination was set up at each location to examine the dogs. Additionally, dogs at veterinary clinics or pre-euthanasia at the municipal from those areas were included.

### Blood collections

Blood samples from a total of 526 dogs (279 males and 247 females) of varying ages (0≤1 yr to >1 yr old) were collected; a sample of 2–5 ml from each dog from the cephalic vein into EDTA vacuum tubes (BD Vacutainer® Tube, Gribbles Pathology, VIC, Australia) and transported to the parasitological laboratory, Shaqra University for DNA extraction.

### Biopsy tissue collections and parasites cultures

Of 526 dogs, forty dogs were suspected for CL and diagnosed clinically by cutaneous nodules or ulcerated lesions present on skin. Skin biopsies of diameter 5mm were taken under sterile conditions from the border of the ulcer, inoculated into medium M199 supplemented (Gibco, Life Technologies, Germany) with 25 mmol/L HEPES (pH:7.5) and 20% fetal bovine serum (Gibco, Germany) followed by incubation (24 °C). Ten days later, parasites were harvested, washed with ice-cold phosphate-buffered saline (PBS, pH: 7.4) and stored in −20 °C before DNA isolation.

### Blood and parasites DNA extraction

DNA from parasite cultures and lesion biopsies was isolated by overnight lysis in NET buffer containing Proteinase K (Sigma) and 1% sodium dodecyl sulfate as described previously (26). For blood samples, a total genomic DNA (gDNA) was isolated using DNeasy Blood and Tissue kit (Qiagen, Hilden, Germany), eluted in 50/100 μl of elution buffer as per manufacturer’s instruction. An aliquot (50 μl − 100 μl of gDNA from each sample) was stored at −80 °C prior to sending to the Molecular laboratory, Plymouth University, for PCR analysis. There, gDNA was stored at −20 °C for up to 1 month prior to diagnostics.

### Leishmania PCR

For PCR amplification of multiple pathogenic *Leishmania* species in a single reaction, a generic kDNA was initially performed to amplify a ∼145 bp region of kDNA minicircles (27). The samples positive for *L. infantum, L. donovani, L. major* and *L. tropica* by kDNA-PCR were subjected to further rounds using species-specific primers to distinguish *Leishmania* at species level (Table 1).

**Table 1:** Summary of primers used with sequences (5′-3) in the PCR assay

***Primer name***	***Primer sequence (5′-3′)***	***Annealing temperature***	***Specificity***	***Reference***
**RV1/RV2**	F: 5′-CTTTTCTGGTCCCGCGGGTAGG-3′ R: 5′-CCACCTGGCCTATTTTACAC-3′	62 °C	*Leishmania* spp	27
**MC1/MC2**	F: 5′-GTTAGCCGATGGTGGTCTTG-3′ R: 5′-CACCCATTTTTCCGATTTTG-3′	50 °C	*L.infantum*/*donovani*	36
**F/R**	F: 5′- TCGCAGAACGCCCCTACC-3′ R: 5′-AGGGGTTGGTGTAAAATAGGC-3′	60 °C	*L.major* /*L. tropica*	37

PCR reaction was performed in a total volume of 50 μL, containing 10 mM Tris-HCl, (pH 9.0), 1.5 mM MgCl2, 50 mM KCl, 200 μM of each deoxynucleotide triphosphate, 1.5 units of Dream Taq DNA polymerase, (Thermo Scientific™, Nalgene, UK), 1 μM of each related primer (RV1/RV2, MC1/MC2 and F/R), and 2 μL of template DNA (10–20 ng of DNA). The reaction was brought to 50 μL total volume with PCR grade water (Invitrogen, Paisley, UK). *Leishmania* positive controls were used as reference strains, including; *L. tropica* (MHOM /Sudan/ 58 OD strain)*, L. major* (MHOM/IL/67/Jericho-II), *L. infantum* (MHOM/ TN/80/IPT1). Reactions were performed in automated thermal cycler (Veriti; Applied Biosystems, Foster City, USA) and consisted of an initial denaturation step (94 °C/10 min), then 45 cycles of denaturation (95 °C/1 min), primer annealing (62 °C/1.5 min), with an exception for MC1/MC2 primers (50 °C/1.5 min) and F/R primers (62 °C/1 min) and primer extension (72 °C/30 sec). A final extension (72 °C/10 min) was performed and samples held at 4°C. Aliquots of 15 μL PCR product were electrophoresed on a 1.7% agarose gel containing 1μL/mL Syber safe (Thermo Scientific™, UK) in Tris-acetate–EDTA buffer (100V/45 min) and visualized under UV imaging system (ImageQuant Laz4000, GE Healthcare Life Science, UK). The size of each product (e.g.: kDNA minicircles region for *L. infantum/donovani* complex =447 bp) was estimated by comparison with Gene Ruler 100 bp DNA Ladder Marker (Thermo Scientific™, UK).

### Sequencing of Leishmania kDNA

*Leishmania* species were determined by sending positive samples to Macrogen Europe (Netherlands) to sequence kDNA minicircles using forward primers. Results were compared with sequences available at GenBank database using BLAST (https://blast.ncbi.nlm.nih.gov/Blast.cgi). The data will be uploaded onto GenBank for future studies.

### Statistical analysis

Statistical analyses were performed with SPSS package (ver. 17.0; IBM, New York, USA). The relationship between infection rates and risk factors, such as gender and age, was analyzed using a chi-squared test of significant *P*-values (*P*<0.05).

## Results

Forty dogs had thick cutaneous lesions (1.5×3 cm) in areas such as mouth, nose, ear, muzzle, abdomen, legs, and between fingers. Ulceration in the left hind foot was noted in a few dogs. Four dogs’ skin lesions were associated with dermatitis, splenomegaly and lymphadenomegaly. Parasite culture was used to diagnose cutaneous leishmaniasis, identified in 31/40 (77.5%) of confirmed positive samples.

**Fig. 2:**
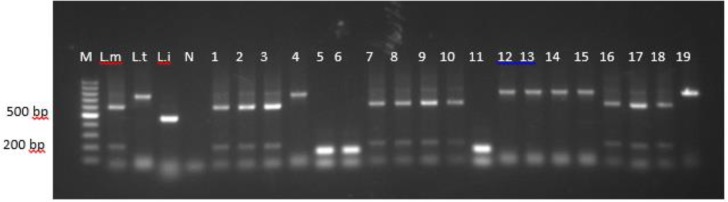
Agarose gel electrophoresis (1.7%) of amplified DNA from collected samples using F/R. M, 100 bp molecular size marker (Generuler); Lane L.m, *L. major*-positive control DNA (200 bp and 620bp); L.t *L. tropica*-positive control DNA (738bp); Li, *L. infantum*-positive control DNA (447bp); N, negative PCR control (water); Lanes1–19, template DNA isolated from stray dog tissue samples from areas during this study shows positive infection with *L. major* and *L. tropica* and negative infection with *L. infantum*

Results obtained from PCR using the RV1/RV2 primer set showed 31 (5.9%) of 526 samples were positive for genus *Leishmania*. Two of 198 Riyadh dogs identified as infected with *Leishmania* spp., with 18 of 175 and 11 of 153 found positive in Al-Ahsa Oasis and Al-Qaseem, respectively. Further PCR analysis using MC1/MC2 primers revealed negative infection with the *L. infantum/donovani* complex. Additional PCR analysis using F/R primers confirmed positive infection for *L. major* with two amplification bands (∼200 bp and ∼620 bp respectively), and *L. tropica* in predictable amplification at ∼738 bp (Fig. 2).

Blast results showed kDNA sequences of *L. major* and *L. tropica -* identified from cutaneous lesions - of 99.9% and 99.8%, similar to Pakistan *L. major* and Iraq *L. tropica* kDNA with accession numbers HQ727556.1 and MF166799, respectively. The results showed Al-Ahsa Oasis as the most endemic area, with prevalence of *L. major* (6.3%) and *L. tropica* (4%). Al-Qaseem was second: *L. major* (5.2%), *L. tropica* (2%) (Table 2). Male dogs were most infected with *L. major* (5.2%), whereas female dogs were most infected with *L. tropica* (2.4%). Moreover, most infections with *L. major* and *L. tropica* were in dogs over one year old (8.1% and 4.5%, respectively). Dog gender and age had significant effects on parasite prevalence: (*P*=0.0212 and 0.0357), respectively (Table 3).

**Table 2:** Prevalence of *Leishmania* species infecting stray dogs in different regions of Saudi Arabia

***Location***	***Leishmania spp.***					

	***L. major***		***L. tropica***		***L. infantum***	
	***Positive No (%)***	***Negative (%)***	***Positive No (%)***	***Negative (%)***	***Positive No (%)***	***Negative (%)***
Riyadh	2 (1.0)	196 (99)	0	198 (100)	0	198 (100)
Al-Ahsa Oasis	11 (6.3)	164 (93.75)	7 (4.0)	168 (96.0)	0	175 (100)
Al-Qaseem	8 (5.2)	145 (94.8)	3 (2.0)	150 (98.0)	0	153 (100)
Total	21 (4.0)	505 (96.0)	10 (1.9)	516 (98.1)	0	526 (100)

**Table 3:** Prevalence and Risk factors associated with *Leishmania* spp. infection among stray dogs in different regions of Saudi Arabia

***Risk Factor***	***Leishmania* spp.**						***P-value***

	***L. major***		***L. tropica***		***L. infantum***		
	***Positive No (%)***	***Negative (%)***	***Positive No (%)***	***Negative (%)***	***Positive No (%)***	***Negative (%)***	
Gender							0.02
Male	16 (5.7.)	263 (94.2)	4 (1.4)	275 (98.6)	0	279 (100)	
Female	5 (2.0)	242 (98.0)	6 (2.4)	241 (97.6)	0	247 (100)	
Total	21 (4.0)	505 (96.0)	10 (1.9)	516 (98.1)	0	526 (100)	
Age group							0.03
≤1 year	3 (1.0)	301 (99.0)	0	304 (100)	0	304 (100)	
>1 year	18 (8.1)	204 (91.9)	10 (4.5%)	212 (95.5)	0	222 (100)	
Total	21 (4.0)	505 (96.0)	10 (1.9)	516 (98.1)	0	526 (100)	

## Discussion

This report was the country’s first extensive molecular and epidemiological study describing dog infections with *L. major* and *L. tropica*, common agents of cutaneous leishmaniasis in Saudi Arabian and Middle Eastern people (2, 15–17).

The study showed cutaneous lesions are the most common manifestation of leishmaniasis in dogs, in agreement with previous studies (28–30). These dogs had cutaneous involvement in different areas. However, the diagnosis of canine leishmaniasis based on clinical features is difficult, needing specific laboratory tests (31,32). Due to few reports of *L. major* and *L. tropica* infections in dogs, more investigations are needed to confirm accurate clinical signs, standardized serological/hematological methods, and chemical/biochemical parameters. We did not attempt histopathological and microscopic examinations due to the limitation of this study.

Several biochemical, histopathological and serological methods have been used to differentially diagnose *Leishmania* species, but there are drawbacks: being intensive, time-consuming, and requiring estimating 10–20 enzymes and specific parasite isolating media (33). PCR assays have been proven as appropriate tools for *Leishmania* species identification (34). However, in endemic regions where more than one *Leishmania* species exists, diagnostic tools are required to detect and distinguish all suspected cases at species level (35).

In this study, electrophoresis data displayed a product size of approximately 145 bp for RV1/RV2 and 620 bp and 780 bp for F/R primers pairs regions of the kDNA of *Leishmania* spp and of the *L. major* and *L. tropica*, respectively. MC1/MC2 primers pairs were used to amplify 447 bp of the *L. infantum/donovani* complex. These primers were used in differential molecular diagnosis of *Leishmania* species causing visceral and cutaneous leishmaniasis (36–39). Targeting a region of kDNA minicircles appeared effective for detecting *Leishmania* spp. because of high copy numbers, variability in amplicon size and detection ability <1pg of *Leishmania* parasite DNA (40–42).

Overall, prevalence of dog CL in these regions was determined at 31/526 dogs (21 cases *L. major* and 10 cases *L. tropica*). Two previous Saudi studies that reported natural infections of *L. major* in dogs using enzymatic biochemical methods (19, 23), but no clinical information was available, no serology was performed or molecular confirmation done as it was unavailable hence, no treatment has been reported. To our knowledge, few studies have determined *L. major* as a cause of cutaneous manifestations in dogs; though Egyptian, Iranian, and Iraqi studies have confirmed infection with *L. major* by enzymatic biochemical and serological methods and molecular tools (20, 21, 36, 38). *L. tropica* infection in dogs has been reported in few countries such as Morocco (43) and Iran (16,44).

This study found all dogs’ samples were negative of *L. infantum* infection in examined areas. Earlier studies reported *L. infantum* has been endemic in the South of Saudi Arabia but has not been reported in the other parts of the country (13,14), suggesting that dogs could be reservoirs of visceral leishmaniasis in this region. However, *L. infantum -* spread through *Phlebotomus* species with dogs acting as reservoirs - are the largest source of human *L. infantum* infection (45, 46). However, in Saudi Arabia, the role of *Phlebotomus* species in VL transmission is still unknown and surveys that are more molecular are needed.

Our results showed that gender and age significantly affect the prevalence of canine *Leishmania* infection, with males more infected than females (*P*>0.0212) and adults more than younger dogs (*P*>0.0357). Other studies reported dogs under a year old being infected with *L. major* (22, 29) and both young infected with *L. major* and *L. tropica* (16, 30) and dogs over 5 yr old infected with *L. tropica (*45). The higher leishmaniasis in male and adult dogs in this study might be due to primarily examining males, or attributed to several factors such as dog migration or traveling to regions where flies are common*,* poor management or body condition leading to weak immunity, stress, and fly preference. However, investigations that are more molecular are required on domestic and wild animals infected with *L. major* and *L. tropica* may be more prevalent in endemic areas. Moreover, epidemiological surveys are needed on the role of *Phlebotomus* species cutaneous and visceral leishmaniasis transmission in Saudi Arabia.

## Conclusion

This is a first molecular study of dog leishmaniasis from Saudi Arabia confirmed to have cutaneous leishmaniasis caused by *L. major* and *L. tropica*. The relationship between the disease vectors and reservoirs with transmission cycle in endemic areas of Saudi Arabia still unknown. Further epidemiological and molecular investigations are required for implementation of future cutaneous leishmaniasis control programs.
